# Cross-country analysis of strategies for achieving progress towards global goals for women’s and children’s health

**DOI:** 10.2471/BLT.15.168450

**Published:** 2016-05-02

**Authors:** Syed Masud Ahmed, Lal B Rawal, Sadia A Chowdhury, John Murray, Sharon Arscott-Mills, Susan Jack, Rachael Hinton, Prima M Alam, Shyama Kuruvilla

**Affiliations:** a Centre of Excellence for Universal Health Coverage, James P Grant School of Public Health, BRAC University, Dhaka, Bangladesh.; b Health Systems and Population Studies Division, International Center for Diarrhoeal Diseases Research, Bangladesh (ICDDR,B), Dhaka, Bangladesh.; c Independent Expert and Consultant Reproductive, Maternal and Child Health and Nutrition Policy and Implementation, Dhaka, Bangladesh.; d Maternal and Child Health Consultant, Iowa City, United States of America (USA).; e International Health and Development, ICF International, Fairfax, USA.; f Centre for International Health, University of Otago, Dunedin, New Zealand.; g Partnership for Maternal, Newborn & Child Health, Geneva, Switzerland.; h Family, Women’s and Children’s Health, World Health Organization, Avenue Appia 20, 1211 Geneva 27, Switzerland.

## Abstract

**Objective:**

To identify how 10 low- and middle-income countries achieved accelerated progress, ahead of comparable countries, towards meeting millennium development goals 4 and 5A to reduce child and maternal mortality.

**Methods:**

We synthesized findings from multistakeholder dialogues and country policy reports conducted previously for the Success Factors studies in 10 countries: Bangladesh, Cambodia, China, Egypt, Ethiopia, the Lao People's Democratic Republic, Nepal, Peru, Rwanda and Viet Nam. A framework approach was used to analyse and synthesize the data from the country reports, resulting in descriptive or explanatory conclusions by theme.

**Findings:**

Successful policy and programme approaches were categorized in four strategic areas: leadership and multistakeholder partnerships; health sector; sectors outside health; and accountability for resources and results. Consistent and coordinated inputs across sectors, based on high-impact interventions, were assessed. Within the health sector, key policy and programme strategies included defining standards, collecting and using data, improving financial protection, and improving the availability and quality of services. Outside the health sector, strategies included investing in girls’ education, water, sanitation and hygiene, poverty reduction, nutrition and food security, and infrastructure development. Countries improved accountability by strengthening and using data systems for planning and evaluating progress.

**Conclusion:**

Reducing maternal and child mortality in the 10 fast-track countries can be linked to consistent and coordinated policy and programme inputs across health and other sectors. The approaches used by successful countries have relevance to other countries looking to scale-up or accelerate progress towards the sustainable development goals.

## Résumé

**Objectif:**

Déterminer comment dix pays à revenu faible et intermédiaire ont réussi à accomplir des progrès accélérés, vis-à-vis de pays comparables, en vue d'atteindre les objectifs 4 et 5A du Millénaire pour le développement visant à réduire la mortalité infantile et maternelle.

**Méthodes:**

Nous avons synthétisé les conclusions de dialogues multipartites et de rapports d'orientation nationaux précédemment établis pour étudier les facteurs de succès dans les dix pays concernés: le Bangladesh, le Cambodge, la Chine, l'Égypte, l'Éthiopie, le Népal, le Pérou, la République démocratique populaire lao, le Rwanda et le Viet Nam. Une approche de cadre a été utilisée pour analyser et synthétiser les données tirées des rapports nationaux, ce qui a donné lieu à des conclusions descriptives ou explicatives en fonction de différents thèmes.

**Résultats:**

Les approches politiques et de programme réussies ont été classées dans quatre domaines stratégiques: leadership et partenariats multipartites; secteur de la santé; secteurs autres que celui de la santé; et responsabilisation en matière de ressources et de résultats. Les contributions cohérentes et coordonnées dans les différents secteurs, basées sur des interventions à fort impact, ont été évaluées. Dans le secteur de la santé, les principales stratégies politiques et de programme incluaient la définition de normes, la collecte et l'utilisation de données, l'amélioration de la protection financière et l'amélioration de l'accessibilité et de la qualité des services. En dehors du secteur de la santé, les stratégies impliquaient des investissements dans l'éducation des filles, l'eau, l'assainissement et l'hygiène, la réduction de la pauvreté, la nutrition et la sécurité alimentaire et le développement des infrastructures. Les pays ont amélioré leur responsabilisation grâce au renforcement et à l'utilisation de systèmes de données permettant de planifier et d'évaluer les progrès.

**Conclusion:**

La réduction accélérée de la mortalité maternelle et infantile dans les dix pays peut être liée à l'élaboration de politiques et de programmes cohérents et coordonnés au sein du secteur de la santé et des autres secteurs. Les approches utilisées par les pays qui enregistrent des succès présentent un intérêt pour d'autres pays cherchant à augmenter ou à accélérer les progrès en vue des objectifs de développement durable.

## Resumen

**Objetivo:**

Identificar el modo en que diez países con ingresos bajos y medios han logrado un progreso acelerado, por delante de países comparables, camino de cumplir los objetivos de desarrollo del milenio 4 y 5A para reducir la mortalidad infantil y materna.

**Métodos:**

Se sintetizaron los resultados de diálogos entre varias partes y los informes de políticas de países redactados anteriormente en relación con los estudios de los factores de éxito en los diez países: Bangladesh, Camboya, China, Egipto, Etiopía, Nepal, Perú, República Democrática Popular Lao, Rwanda y Viet Nam. Se utilizó un enfoque de marco para analizar y sintetizar los datos de los informes de países, lo que dio lugar a conclusiones descriptivas o explicativas por tema.

**Resultados:**

Se categorizaron los enfoques de programas y políticas que tuvieron éxito en cuatro áreas estratégicas: alianzas de liderazgo y de diversas partes interesadas; sector sanitario; sectores no sanitarios; y contabilidad de recursos y resultados. Se evaluaron las contribuciones coherentes y coordinadas en todos los sectores, en base a las intervenciones con gran impacto. Dentro del sector sanitario, las estrategias fundamentales de programas y políticas incluyeron la definición de estándares, la recopilación y el uso de datos, la mejora de la protección financiera y la mejora de la disponibilidad y calidad de los servicios. Fuera del sector sanitario, las estrategias incluyeron la inversión en la educación de niñas, agua, saneamiento e higiene, reducción de la pobreza, nutrición y seguridad alimentaria, y desarrollo de infraestructuras. Los países mejoraron su contabilidad consolidando y utilizando sistemas de datos para el progreso de planificación y evaluación.

**Conclusión:**

La reducción de la mortalidad materna e infantil en los diez países de vía rápida puede estar relacionada con las contribuciones coherentes y coordinadas de programas y políticas, tanto en el sector sanitario como en otros. Los enfoques utilizados por los países que han tenido éxito son relevantes para otros países que buscan aumentar y acelerar el progreso hacia los objetivos de desarrollo sostenible.

## ملخص

**الغرض:**

تحديد كيفية تفوق 10 من البلدان منخفضة الدخل والبلدان متوسطة الدخل على الدول النظيرة لها في تحقيق التقدم الحثيث نحو تنفيذ الأهداف 4 و5أ من الأهداف الإنمائية لهذه الألفية من أجل الحد من وفيات الأمهات والأطفال.

**الطريقة:**

عملنا على توليف النتائج التي أسفرت عنها الحوارات التي تشمل العديد من الجهات المعنية وكذلك التقارير التي سبق إعدادها بشأن السياسة التي تنتهجها الدول للخروج بدراسات تتناول عوامل النجاح في تلك الدول العشرة: بنغلاديش، وكمبوديا، والصين، ومصر، وإثيوبيا، وجمهورية لاو الديمقراطية الشعبية، ونيبال، وبيرو، ورواندا، وفييت نام. وتم استخدام نهج إطاري لتحليل وتوليف البيانات المستمدة من التقارير التي تتناول البلدان، مما أثمر عن استنتاجات وصفية أو توضيحية مصنفة حسب الموضوع.

**النتائج:**

تم تصنيف الأساليب المتبعة لتطبيق السياسات والبرامج الناجحة إلى أربعة مجالات استراتيجية: شراكات بين القيادات والجهات المعنية؛ وقطاع الصحة؛ والقطاعات خارج نطاق الصحة؛ وتحمل المسؤولية عن الموارد والنتائج. وتم تقييم مدخلات تتسم بالاتساق والتنسيق فيما بين العديد من القطاعات بناءً على التدخلات بالغة الأثر. وتضمنت الاستراتيجيات الرئيسة للسياسات والبرامج التي يشملها قطاع الصحة تحديد المعايير، وجمع البيانات واستخدامها، وتعزيز الحماية المالية، وتحسين مدى توفر الخدمات وجودتها. وفيما يتعلق بالقطاع خارج نطاق الصحة تضمنت الاستراتيجيات الاستثمار في مجال تعليم الفتيات، والمياه، والنظافة والعادات الصحية، والحد من الفقر، والتغذية والأمن الغذائي، وتطوير البنية التحتية. ورفعت الدول من مستوى تحمل المسؤولية عن طريق تعزيز نظم البيانات وتفعيلها للتخطيط وتقييم مدى إحراز التقدم.

**الاستنتاج:**

إن الحد من وفيات الأمهات والأطفال في الدول العشرة سريعة التقدم على ذلك الطريق يمكن ربطه باتباع سياسة تتسم بالاتساق والتنسيق وكذلك مدخلات البرامج عبر العديد من القطاعات التي تشمل قطاع الصحة وغيره من القطاعات. وتتصل الأساليب التي تتبعها الدول الناجحة بدول أخرى تتطلع إلى التقدم بمعدل أعلى أو وتيرة أسرع نحو تحقيق أهداف التنمية المستدامة. ****

## 摘要

**目标:**

为了确定 10 个低收入和中等收入国家如何取得加速进展，领先于同等水平的国家，实现千年发展目标 4 和 5A，以及降低儿童死亡率和产妇死亡率。

**方法:**

我们综合了之前在10 个国家的成功要素研究中开展的多方利益相关者对话以及国家政策报告中的研究结果： 这 10 个国家是：埃及、埃塞俄比亚、柬埔寨、老挝人民民主共和国、卢旺达、孟加拉国、秘鲁、尼泊尔、越南和中国。 采用框架法来分析和综合来自国家报告的数据，依照主题生成描述性或解释性结论。

**结果:**

成功的政策和计划方法分为四个战略领域： 领导阶层和多方利益相关者的伙伴关系；卫生部门；卫生以外的部门以及资源和成果问责制。 基于极具影响力的干预措施，对各部门的一致性和协调性投入进行了评估。 在卫生部门，主要政策和计划策略包括定义标准、收集和使用数据、提高财务保障以及提高服务的可获得性和质量。 在卫生部门以外，战略包括女童教育投资、供水、环境卫生和个人卫生、减少贫困、营养和食品安全以及基础设施发展。国家通过加强和使用数据系统进行规划和评估进展以改善问责制。

**结论:**

在 10 个快速发展国家，可以将降低产妇死亡率和儿童死亡率与卫生部门和其他部门的一致性和协调性政策和计划投入相结合。 成功国家采用的方法可以与其他国家相关联，以寻求按比例增加或加速实现可持续发展目标的进展。

## Резюме

**Цель:**

Определить, как 10 странам с низким и средним уровнем доходов удалось продемонстрировать ускоренный по сравнению с сопоставимыми странами прогресс в плане достижения целей тысячелетия 4 и 5A в области развития, сформулированных в Декларации тысячелетия, предполагающих сокращение детской и материнской смертности.

**Методы:**

Данные, полученные из ранее проведенных диалогов с участием широкого круга заинтересованных сторон и ранее составленных отчетов о стратегических направлениях развития стран, были обобщены для изучения факторов успеха в 10 странах: Бангладеш, Вьетнам, Египет, Камбоджа, Китай, Лаосская Народно-Демократическая Республика, Непал, Перу, Руанда и Эфиопия. С помощью рамочного подхода данные из докладов стран были проанализированы и обобщены, в результате чего были сделаны выводы по теме с описаниями или объяснениями.

**Результаты:**

Успешные подходы к воплощению планов действия и программ были отнесены к четырем стратегическим областям: руководство и партнерство с большим количеством заинтересованных сторон, сфера здравоохранения, другие сферы экономики и ответственность за ресурсы и результаты. Была проведена оценка систематического и согласованного вклада из различных секторов, в основу которого были заложены высокоэффективные мероприятия. В сфере здравоохранения основные стратегии планов действий и программ включали определение стандартов, сбор и применение данных, повышение финансовой защиты, а также доступности и качества услуг. В других сферах стратегии включали инвестирование в системы образования для девочек, водоснабжение и область санитарии и гигиены, сокращение масштабов бедности, обеспечение продовольственной безопасности и безопасности питания и развитие инфраструктуры. Усиление механизмов подотчетности в странах было достигнуто за счет совершенствования и использования систем данных для планирования и анализа хода развития.

**Вывод:**

Снижение уровней материнской и детской смертности в 10 странах, демонстрирующих ускоренный прогресс, может быть связано с систематическим и согласованным вкладом, внесенным в результате реализации стратегических планов и программ, в сферу здравоохранения и другие сферы. Подходы, которые использовали достигшие успеха страны, могут быть применены в других странах, стремящихся ускорить продвижение по пути к достижению целей в области устойчивого развития.

## Introduction

Between 1990 and 2015, during the era of the millennium development goals (MDGs), there was unprecedented global progress towards reducing both child and maternal mortality by around 50%.^1^^,^^2^ Progress was uneven, however, between and within countries. Of the 95 countries with maternal mortality ratios above 100 deaths per 100 000 live births in 1990, nine countries achieved MDG 5A to reduce maternal mortality by three quarters. Only 24 out of 104 low- and middle-income countries met the MDG 4 target of a two-thirds reduction in the under-five mortality rate between 1990 and 2015.^3^^,^^4^ To understand why some countries did better than other comparable countries in preventing maternal and child deaths, a three-year multidisciplinary, multi-country series of studies on success factors for women’s and children’s health, referred to as the Success Factors studies, was undertaken.^4^

Among the 75 highest-burden countries flagged up by the Countdown to 2015 initiative,^3^ 10 low- and middle-income countries were on track to achieve both MDGs 4 and 5A when the Success Factors studies started in 2012: Bangladesh, Cambodia, China, Egypt, Ethiopia, the Lao People's Democratic Republic, Nepal, Peru, Rwanda and Viet Nam.^5^ Focusing on what contributed to the higher reduction of maternal and child mortality rates in these countries, the studies identified an integrated set of high-impact factors in the health sector and sectors outside health, underpinned by strong country leadership, collaboration between different stakeholders and economic development.^5^ Statistical, econometric and policy analyses showed that these countries were not only progressing faster on mortality reductions, but were also performing significantly better than comparable countries on the identified success factors.^5^

Based on the initial analyses of success factors, we conducted a series of multistakeholder dialogues in the 10 fast-track countries to identify how these countries designed and implemented policies and programmes in the areas identified as success factors. This paper presents a synthesis of the multistakeholder dialogue findings across the countries. These findings informed the development of the *Global strategy for women’s, children’s and adolescents’ health (2016–2030)*^6^ and could inform country policies and programmes to help accelerate progress towards meeting the sustainable development goals (SDGs).

## Methods

The first part of the success factors studies comprised comparative analyses of data from 144 low- and middle-income countries over 20 years and a literature review of countries’ progress in reducing maternal and child mortality during the MDG period, as described before.^4^^,^^5^ Subsequently, between 2014 and 2015, country policy reports were developed through multistakeholder dialogues in the 10 fast-track countries identified in the Success Factors studies.^4^ A multistakeholder dialogue is a structured, facilitated process that brings stakeholders together to develop a shared understanding of issues and evidence and to develop plans of action. In total, 407 stakeholders (representatives of government, academia, civil society, private sector, multilateral and other development partner organizations) across the 10 countries took part in the dialogues. Each dialogue was conducted in three phases: (i) preparation and review of literature and data; (ii) discussion meetings, usually over two days, supplemented in some cases by one-to-one interviews and additional meetings; and (iii) validation and preparation of country reports and dissemination of findings. The methods for the multistakeholder dialogues are described in more detail elsewhere.^7^ Ethics approval for the Success Factors studies was obtained from the World Health Organization (WHO) Ethics Review Committee (reference RPC528), and participants in the dialogues gave consent to be interviewed for the analysis.

To analyse and synthesize the data from the 10 country reports resulting from the dialogues,^4^ we adapted and used the Framework Method as it is appropriate for comparing and contrasting large-scale textual data across cases.^8^ This method comprises seven steps: (i) transcription; (ii) familiarization with the data; (iii) coding; (iv) developing a working analytical framework; (v) applying the analytical framework; (vi) inserting data into the framework matrix; and (vii) interpreting the data. Similarities and differences in the data can be identified and relationships drawn across different parts of the analysis, resulting in descriptive or explanatory conclusions by theme.^9^

Transcription of country policy reports, meeting documents and stakeholder interviews was completed during the dialogue process in the countries.^7^ The cross-country analysis started with examining the data and initial coding. We developed a modified health systems framework matrix which was populated with data from the country policy reports and categorized by the main strategic areas where policy and programme inputs had been made.^7^^,^^10^ We further coded the data to identify common themes, focusing on key policies and programmes and strategic areas, until we identified no new themes.^11^ Based on this approach, we synthesized the countries’ policy and programme approaches into strategic areas. We triangulated findings, where possible, with related literature reviews and other data.^4^

## Results

From 1990 to 2015 the countries achieved major reductions in under-five child mortality (Table 1) and maternal mortality (Table 2) and there were associated improvements in population-based coverage of high-impact interventions in health and other sectors. Stakeholders in the 10 countries identified policies and programmes that contributed to this progress, and the review of data between 1990 and 2015 highlighted related trends under four strategic areas: leadership and multistakeholder partnerships; health sector; sectors outside health; and accountability for resources and results (Table 3 and Table 4). Table 5 presents additional examples of country policies and programmes that were identified in the dialogues as contributing to progress towards MDGs 4 and 5A; further details are found in the country reports and their web annexes.^13^

**Table 1:** Reduction of under-five mortality rate between the years 1990 and 2015 in 10 countries with accelerated progress towards reducing child and maternal mortality

Country	No. of child deaths per 1000 live births						Decrease 1990–2015, (%)	Annual rate of reduction, %
	1990	1995	2000	2005	2010	2015		
Bangladesh	144	114	88	67	49	38	106 (73.8)	5.4
Cambodia	117	122	108	64	43.8	29	88 (75.5)	5.6
China	54	48	37	24	15.8	11	43 (80.1)	6.5
Egypt	86	64	47	31	24	24	62 (72.1)	5.1
Ethiopia	205	175	145	110	76	59	146 (71.1)	5.0
Lao People's Democratic Republic	162	140	118	97	80	67	95 (58.9)	3.6
Nepal	141	109	81	60	45	36	105 (74.5)	5.5
Peru	80	58	39	28	20	17	63 (78.8)	6.2
Rwanda	152	253	184	106	63.6	42	110 (72.5)	5.2
Viet Nam	51	42	34	30	26	22	29 (57.2)	3.4

Note: Under-five mortality rate is number of deaths of children aged 0–4 years in a given period per 1000 live births in the same period.

Sources**:** UN Inter-agency Group for Child Mortality Estimation^1^ and World Bank.^12^

**Table 2:** Reduction of maternal mortality ratio between the years 1990 and 2015 in 10 countries with accelerated progress towards reducing child and maternal mortality

Country	No. of maternal deaths per 100 000 live births						Decrease 1990–2015, (%)	Annual rate of reduction, %
	1990	1995	2000	2005	2010	2015		
Bangladesh	569	479	399	319	242	176	393 (69.1)	4.7
Cambodia	1020	730	484	315	202	161	859 (84.2)	7.4
China	97	72	58	48	35	27	70 (72.2)	5.2
Egypt	106	83	63	52	40	33	73 (68.9)	4.7
Ethiopia	1250	1080	897	743	523	353	897 (71.8)	5.0
Lao People's Democratic Republic	905	695	546	418	294	197	708 (78.2)	6.1
Nepal	901	660	548	444	349	258	643 (71.4)	5.0
Peru	251	206	140	114	92	68	183 (72.9)	5.2
Rwanda	1300	1220	1020	567	381	290	1010 (77.7)	6.0
Viet Nam	139	107	81	61	58	54	85 (61.1)	3.8

Note: Maternal mortality ratio is the number of deaths of women while pregnant or within 42 days of termination of pregnancy in a given period per 100 000 live births in the same period.

Sources**:** WHO, UNICEF, UNFPA, World Bank Group and the United Nations Population Division^2^ and World Bank.^12^

**Table 3:** Key policy and programme actions in four strategic areas identified as important for accelerated progress towards reducing child and maternal mortality

Strategic area	Policy and programme actions	Illustrative performance measures
Leadership and multistakeholder partnerships	Develop policies, strategies, plans and mechanisms to guide programme implementation by: – establishing mechanisms for coordination and collaboration within government and between government and partners; – strengthening governance by reducing corruption, maintaining transparency and accountability, and improving civic participation and representation of women in government; – collecting data for planning, including research and innovation; – defining interventions that will be delivered by the programme along the lifecycle of women and children, with a focus on high-impact, evidence-based interventions; – developing or revising policies, standards and guidelines that are informed by human rights, and supporting implementation of priority interventions that are evidence-based and target high-risk groups; and – developing short- and long-term plans to deliver priority interventions.	– Rule of law – Government effectiveness –Women in parliament – Female labour force participation
Health sector	Strengthen essential health systems to deliver priority interventions by: – deciding how interventions will be delivered; – ensuring adequate resources are available and removing financial barriers to accessing health care; – ensuring availability of trained staff; – strengthening quality of and demand for care; – increasing the number of primary health care and specialized maternal and child care centres; and – ensuring supply of high-quality essential medicines and commodities.	– Births assisted by skilled staff – Physicians per population – Total fertility rate – Immunization (DTP and measles)
Sectors outside health	Strengthen sectors which support improved health and nutrition by: – increasing financial resources allocated to key sectors, targeted to areas with highest morbidity and mortality; – investing in infrastructure to improve transportation and communication; – promoting education of girls and poor people; enhancing training and deployment of teachers, particularly women; – developing water and sanitation initiatives with communities; – developing information and communication technologies, including e-health initiatives; – improving income-generation opportunities for poor people; and – developing cross-sectoral approaches to improve nutrition.	– Clean water supply – Access to sanitation – Primary-school enrolment (female and total) – Secondary-school enrolment (female and total) – Roads paved – Rural electricity
Accountability for resources and results	Collect and use data for planning and evaluating progress by: – adopting standard indicators and targets; – strengthening routine health management information system, incorporating key indicators and vital registration; – conducting regular performance reviews; – conducting maternal and neonatal death audits; – improving access to electronic data collection and management systems at all levels including Internet databases, and use of SMS tracking systems.	– GDP per capita – Gini index – Total health expenditure

DTP: diphtheria–tetanus–pertussis; GDP: gross domestic product; SMS: short message service.

**Table 4:** Changes in development indicators from 1990–1995 and 2010–2015 across four key strategic areas in 10 countries with accelerated progress towards reducing child and maternal mortality

Development indicator^a^ by strategic area	Bangladesh		Cambodia		China		Egypt		Ethiopia		Lao People's Democratic Republic		Nepal		Peru		Rwanda		Viet Nam	
	1990–1995	2010–2015	1990–1995	2010–2015	1990–1995	2010–2015	1990–1995	2010–2015	1990–1995	2010–2015	1990–1995	2010–2015	1990–1995	2010–2015	1990–1995	2010–2015	1990–1995	2010–2015	1990–1995	2010–2015
**Leadership and multistakeholder partnerships**																				
Rule of law^b^	−1.0	−0.8	−1.1	−1.1	−0.4	−0.4	0.1	−0.2	−0.9	−0.8	−1.0	−1.0	−0.2	−1.0	−0.7	−0.6	−1.7	−0.4	−0.4	−0.5
Government effectiveness^c^	−0.7	−0.8	−0.9	−0.9	−0.3	0.1	−0.2	−0.4	−1.3	−0.5	−0.7	−0.9	−0.4	−0.9	−0.1	−0.3	−1.2	−0.1	−0.5	−0.3
Women in parliament, % of total seats	9	20	6	22	22	24	–	–	2	28	–	25	–	33	–	26	17	64	26	24
Female labour force, % of total labour force	62	57	77	79	73	64	24	23	72	78	80	76	80	80	45	68	89	87	74	72
**Programme implementation in health sector**																				
Births attended by skilled health staff, % of total	10	28	31	71	94	100	37	74	6	10	19	37	7	36	–	84	31	69	70	93
Physicians, no. per 1000 population	0.2	0.4	0.1	0.2	1.6	1.9	0.8	2.8	0.0	0.0	0.2	0.2	0.1	0.2	1.1	1.1	0.0	0.1	0.4	1.2
Total fertility rate, births per 1000 women aged 15–19 years	5	2	6	3	3	2	4	3	7	5	6	3	5	3	4	3	7	5	4	2
Immunization, DTP, % of children aged 12–23 months	64	96	41	93	95	99	84	97	32	61	20	73	44	88	67	92	86	97	88	95
Immunization, measles, % of children aged 12–23 months	62	91	37	93	95	99	87	96	26	64	33	64	57	88	63	94	85	95	84	97
**Programme implementation in other sectors**																				
Improved water supply, % of population with access	68	87	22	76	67	96	93	94	13	57	40	76	67	92	75	87	61	76	62	98
Improved sanitation facilities, % of population with access	34	90	3	42	25	77	72	95	2	28	20	71	7	46	54	76	31	62	38	78
School enrolment ratio, primary, female, %	71	110	118	126	121	129	84	108	28	78	87	117	84	145	116	106	73	125	104	102
School enrolment ratio, primary, total, %	79	107	115	129	128	129	92	111	35	71	98	122	115	141	118	107	74	123	105	104
School enrolment ratio, secondary, female, %	13	52		30	33	84	60	77	12	25	19	40	21	58	64	90	15	29	–	–
School enrolment ratio, secondary, total, %	20	50	28	35	39	83	69	78	15	19	23	44	35	59	67	91	16	29	34	–
Roads paved, % of total roads	7	–	8	6	–	61	72	91	15	14	20	14	38	56	10	16	9	19	24	45
Rural electricity access, % of rural population	43	49	19	19	98	100	99	100	100	100	52	55	72	72	60	73	4	8	95	98
**Accountability for resources and results**																				
GDP per capita, current US$	268	1087	262	1095	483	7590	867	3199	137	574	260	1793	235	702	1916	6541	239	696	302	2552
Gini index^d^	28.8	32.1	38.3	33.4	32.4	42.1	32.0	–	40.0	33.6	–	–	35.2	32.8	44.9	45.6	–	50.8	–	39.3
Health expenditure, total, % of total GDP	5.5	3.7	5.3	7.5	3.5	5.6	3.9	5.1	3.0	5.1	4.1	2.0	5.3	6.0	4.6	5.3	4.3	11.1	5.2	6.0

DTP: diphtheria–tetanus–pertussis; GDP: gross domestic product; US$: United States dollars.

^a^ World development indicators from the World Bank Group.^12^

^b^ Rule of law captures perceptions of the extent to which agents have confidence in and abide by the rules of society, and in particular the quality of contract enforcement, property rights, the police, and the courts, as well as the likelihood of crime and violence.

^c^ Government effectiveness captures perceptions of the quality of public services, the quality of the civil service and the degree of its independence from political pressures, the quality of policy formulation and implementation, and the credibility of the government's commitment to such policies.

^d^ The Gini index measures the extent to which the distribution of income (or, in some cases, consumption expenditure) among individuals or households within an economy deviates from a perfectly equal distribution.

Note: The closest year was used as data were not available for all 10 countries in all years, as indicated by the dashes.

**Table 5:** Examples of policy and programme actions towards development successes of 10 countries with accelerated progress towards reducing child and maternal mortality

Country	Leadership and multistakeholder partnerships	Health sector	Sectors outside health	Accountability for resources and results
Bangladesh	Led multi-country evaluation of integrated case management of childhood illness, leading to global scale-up of initiative.	Since 1973 country is committed to expanding rural health infrastructure to provide comprehensive services.	Strategic planning increased the number of paved roads from 9704 to 17 321 over 1991–2007.	Provided wireless Internet and laptops to 12 527 community clinics and 4500 union health centres.
Cambodia	Based planning on research findings into causes of newborn and child deaths; micronutrient sprinkles (single-dose supplement packets); and health financing schemes.	Developed a midwifery training and incentive scheme. By 2013, 75% of health facilities had at least one secondary midwife (with at least 3 years of basic training).	Increased % of government spending on infrastructure from 4.7% in 2004 to 9.5% in 2010.	Since 2011 health information system data demonstrate a 99% rate of reporting completeness and data internal consistency.
China	Established a policy and legal framework through a law on maternal and infant child care and National Programme for Women’s and Children’s Development.	Used target responsibility agreements with service providers, for example to improve quality of care for immunization and antenatal care and achieve the related national targets.	Introduced compulsory universal free education for the first 9 years, and initiatives to improve school access for the underserved.	Implemented a comprehensive surveillance and health information system.
Egypt	Used data on levels and causes of maternal mortality for planning strategies. Created national councils to strengthen rural women’s participation in health and development.	*Fatwa* (Islamic religious ruling) issued by the Grand Mufti of Egypt in support of family planning aimed to change traditional society views. Fertility dropped from 5 children per woman in 1980 to < 3 in 2011.	Invested resources in Upper Egypt to improve access to water and sanitation.	Conducts annual data reviews and planning using routine health information system data. Used data on levels and causes of maternal mortality for planning strategies.
Ethiopia	Created a government department to support development in pastoralist areas.	Developed new cadres: health extension workers and non-doctor clinicians to improve access to care.	Initiated multi-sectoral coordinated nutrition planning targeting the first 1000 days of life.	Monitors progress in reproductive, maternal, newborn and child health using a routine data scorecard to identify and respond to service gaps.
Lao People's Democratic Republic	Defined minimum package of interventions that must be delivered at each level of the health system.	Abolished user fees so as to improve care-seeking by women and children.	Increased public expenditure on education sixfold.	developed Health information system strategic plan, including institution-based data collection systems and vital registration system.
Nepal	Instituted sector-wide approaches to improve donor coordination focused on national priorities.	Increased the number of health facilities from 975 to 4000, and birthing centres from 422 to 1121.	Pioneered a multisectoral and high-level coordinated national nutrition plan.	Scaled-up maternal death surveillance and reviews.
Peru	Ministry for Women and Social Development promotes social development and equal opportunity for women and excluded groups.	Trained health staff in culturally sensitive emergency obstetric and neonatal care.	Adopted a multisectoral strategy to address poverty and socially excluded groups.	Introduced a perinatal reporting system to track maternal and newborn deaths.
Rwanda	Instituted sector-wide approaches to improve donor coordination and alignment with national priorities.	Introduced 45 000 community health workers to provide essential services.	Developed nationwide Internet access and rapid SMS technology for community health worker reporting.	Instituted a community reporting system for births and for maternal and child deaths.
Viet Nam	Established national technical working groups on reproductive, maternal, newborn and child health.	Routinely updates the essential medicine list and enforces standards to maintain quality.	Invested in commune infrastructure with funding targeted to poor and marginal households.	Published national health data on a website to promote transparency and use.

SMS: short message service.

### Leadership and partnerships

Countries demonstrated leadership by using data from population-based surveys and research to develop policies and plans to reach high-risk populations (Table 3 and Table 5). They prioritized high-impact interventions and used technical standards to guide implementation. Human rights policies helped ensure high-risk groups were protected and prioritized.^10^ In some cases, governments established agencies to support implementation;^14^^,^^15^ for example Egypt created national councils to strengthen rural women’s participation in health and development.^14^

Countries took steps to improve governance by increasing transparency and accountability, reducing corruption and creating opportunities for civic participation (Table 4). Progress varied among countries. In Ethiopia, reforms in governance reduced corruption between 1996 and 2014 and improved the efficiency of the civil service.^16^ Despite political instability, Nepal made modest progress in the rule of law and control of corruption between 2004 and 2014.^17^ The proportion of women members in the national legislature of the Lao People's Democratic Republic tripled between 1990 and 2003, so that by 2014 women comprised a quarter of members of parliament.^18^ Rwanda achieved female representation in 64% of the seats in the parliament and 40% in the senate.^19^

Collaboration across government entities, development partners and nongovernmental and community-based organizations was improved. In Ethiopia, an agreement in 2005 between the government and its development partners guided partner support for the health sector development programme, and led to other agreements related to international health partnerships and joint financing. Several other countries adopted cross-sector approaches to ensure better allocation and use of resources for health (Table 5).^15^^,^^20^^–^^24^

### Health sector

The countries took steps to strengthen essential health systems to deliver priority interventions (Table 3 and Table 5). They ensured a mix of delivery strategies for interventions in women’s and children’s health, based on their current health system capacity and the country context. Key interventions were delivered using a combination of targeted vertical delivery and campaign strategies, complemented by community-based approaches for hard-to-reach populations. They also strengthened routine facility-based services. Bangladesh began with a vertical immunization programme, but later integrated this into the health-care system, with semi-annual campaigns to deliver vitamin A supplements and polio vaccination.^20^ Nepal used targeted child health campaigns and integrated programmes delivered by female community health volunteers to reach communities with limited access to services.^23^^–^^26^

Various mechanisms were used to improve health financing, including increasing the annual per capita expenditure on health, introducing community-based health insurance, minimizing out-of-pocket expenses on health and providing monetary incentives to marginalized populations. Although governments increased their health expenditure (Table 4), most of them remain highly dependent on external funds. China was an exception; government health expenditure per capita increased from 53 United States dollars (US$) in 1995 to US$ 646 in 2013.^27^ China also introduced a medical scheme which covers 95% of the eligible rural population.^28^

Countries developed short- and long-term approaches to address health workforce challenges. Several countries improved midwifery training and used incentives for recruitment and retention of staff, while others used task shifting and community health workers or volunteers to address staff shortages and reach marginalized populations. Cambodia has dramatically improved its rate of skilled birth attendance (Table 4).^29^ The Chinese government trained large numbers of additional health personnel, including village doctors (barefoot doctors) to strengthen primary care delivery.^30^ Peru improved access to emergency obstetric care in rural communities by training health providers to respond better to local beliefs and expectations.^31^

Actions to improve the quality of care have included strengthened supervision and monitoring systems for service delivery, improved referral mechanisms, implementation of accreditation processes and reviews of maternal deaths (Table 5). Testing of community-based management of neonatal sepsis in Nepal has contributed to improving access to life-saving neonatal care.^32^ Rwanda has focused on specialized training for nurses and doctors.^33^ Bangladesh has promoted health and behaviour change strategies that have increased community demand and use of services.^34^ Cambodia has a strategy to promote exclusive breastfeeding and antenatal care.^35^

### Other sectors

In sectors other than health, the 10 fast-track countries applied policy and programme strategies that invested in girls’ education, poverty reduction, food security and infrastructure development (Table 3 and Table 5). Countries made education – primary, extended primary and sometimes secondary – freely available to all. Access to education for girls was prioritized, with a focus on better deployment and living conditions of teachers, especially female teachers, and incentives for poor families to send their children to school. Bangladesh’s female secondary-school stipend project has rapidly expanded secondary schooling for girls since the 1990s; keeping girls in education longer helps to delay marriage and childbearing, which in turn affects maternal mortality.^36^

Countries invested in water and sanitation infrastructure (Table 4) and focused on community-oriented interventions on specific health issues, such as open defecation. Rwanda introduced several community-based initiatives, including hand-washing stations for restaurants, schools and public places.^37^

Another approach common to these countries was poverty reduction, with clear targets to reach women and vulnerable groups, especially in rural and farming communities. In Bangladesh, rapid expansions in the garment industry and in microcredit programmes increased the number of women employed.^38^ Poverty was reduced in Cambodia due to removal of price controls and taxes on rice production, improved rural infrastructure and a higher minimum wage for garment industry workers.^21^^,^^39^

### Accountability

All the 10 countries collected and used data for planning and evaluating progress towards the MDGs (Table 3 and Table 5). They adopted the MDGs, set their own targets and made commitments to measuring and achieving them.^21^^,^^40^ Most countries relied on regular demographic and health surveys to collect high-quality, population-based data to track progress and to inform planning.

Some countries have begun shifting from paper-based routine health information systems to electronic systems with centralized data management. The maternal and child health surveillance system in China is one of the largest centralized networks of its kind.^41^ Rwanda’s web-based applications enables public access to aggregated health information, thereby improving transparency and encouraging wider use of this information.^42^ Egypt also makes aggregated health information publicly available.^43^

Several fast-track countries have had some success with improving the completeness of their birth and death registration systems. Bangladesh established a centralized online birth and death registration system, and succeeded in registering over half of all children younger than 5 years, up from a baseline of 10%.^44^ Viet Nam introduced community reporting systems for births and maternal and child deaths and the Lao People's Democratic Republic strengthened facility death reporting standards.^45^^,^^46^

## Discussion

We analysed policy and programme inputs identified during the multistakeholder dialogues in the 10 countries and found several common characteristics that made progress in women’s and children’s health possible.

Central to progress in all countries was the development of clear policies, strategies and technical standards, led and coordinated by the government. Countries improved coordination, set priorities, developed long-term strategies and held firm to these commitments, demonstrating strong governance at the highest level, as well as a culture of accountability towards improved use of resources.^16^^,^^17^^,^^19^^,^^27^ Improvements in governance were also enabled by a climate of relative political stability, which allowed policies to be maintained consistently over time and progressively improved.

Countries defined indicators, and collected, used and reviewed data for setting priorities and planning. Data were used to establish high-impact interventions that became the foundation of all programmes, policies and guidelines, and this served to maximize their impact.^20^^,^^41^^–^^43^^,^^47^ Countries made steady improvements in the availability of financial and human resources across all sectors. Innovative methods were also used to improve the financial protection of women and children to improve service utilization and prevent catastrophic out-of-pocket health expenditures.^20^^,^^23^^,^^24^

There was increasing commitment to improving access to and availability of health services to a greater share of the population. Investments in infrastructure, with community involvement, served to improve the availability of primary, secondary or tertiary health-care facilities.^7^ Countries also improved the availability of human resources with investments in the training and recruitment of midwives, by task shifting and through building networks of community health workers to provide preventive care, including basic health screening, and, in some cases, case-management of childhood diseases in the community.^7^

Improvements in sectors outside health contributed to around half the reductions in maternal and child mortality across low- and middle-income countries during the MDGs.^4^^,^^48^ In the 10 fast-track countries, these multisector improvements were driven by a variety of policy and programme approaches that invested in girls’ education, water and sanitation, infrastructure development, food security, and poverty reduction policies such as promoting job growth in rural populations and in industries employing women from low-income settings. Such investments also contributed to reducing socioeconomic, geographical and gender disparities.^4^ These findings reiterate the integrated and holistic approach to health and sustainable development promoted by the SDGs.

### Policy implications

By 2015, eight of the 10 countries on track to achieving MDG 4 had done so, and four countries had achieved MDG 5A. How countries can sustain progress was not the focus of this paper, but is an important area for further research. Some initial inferences can, however, be drawn from the analysis of factors affecting countries’ progress and contextual changes since the time of the dialogues, highlighting critical challenges for the SDG era. 

First, the MDGs imposed the same ambitious targets on every country, irrespective of mortality burden, resources and policy potential. Failure to achieve specific targets in 2015 does not negate the high rate of progress over the previous 20 years, and a lack of reliable data meant that progress overall was estimated. Analyses show that country-specific targets can supplement global targets to identify under- or over-performance relative to a country’s potential, and this could be helpful to track progress towards the SDGs.^49^

Second, while countries were initially able to lower average national rates of mortality, a challenge across all the countries was addressing inequitable access to essential, quality services, especially for people in underserved, marginalized and challenging settings. These groups tend to have higher mortality and are exposed to greater health risks, thereby slowing the overall rate of mortality reduction in a country. Further, the specific needs of adolescent girls who might have high-risk pregnancies were not included in the MDGs, nor were issues concerning adolescent heath overall: issues that are central to improving health and achieving the SDGs. Equity was largely under-evaluated during the MDGs; yet equity has to be a critical concern to all countries under the universal scope of the SDG agenda and considering the aim of the global strategy to reach every woman, child and adolescent in every setting.^6^

Third, the epidemiology of under-five child mortality changes as mortality declines, with an increasing proportion of child deaths occurring in the neonatal period. This was another common challenge identified by the countries as a priority focus for the SDG era. Strategies to prevent neonatal deaths require improved quality of delivery and immediate care of babies after delivery, which is still limited in many countries.

Fourth, continued reductions in maternal mortality require sustained investment and improvements in access, coverage and quality of care to both prevent and manage complicated deliveries. This requires sustained investments in the development of the health workforce, health facilities and other health systems, as well as in the roads and transport to access health services, together with a reduction of financial barriers to access. Policies to strengthen health systems, including the availability of a skilled workforce, are often the most difficult to implement and sustain.

Fifth, external factors such as social, economic and environmental shocks have an important impact on how effectively programmes deliver interventions to women and children and these factors may increase the risk of death. Examples include periodic droughts in Ethiopia, the Nepal earthquake of 2015 and political unrest in Egypt and Bangladesh towards the end of the era of the MDGs.

Finally, in tracking progress, the MDGs focused on measuring reductions in maternal and child mortality without linking these to measurements of factors that contribute to mortality reductions – with around 50% of the reductions associated with the health sector and 50% with sectors outside health.^48^ The SDGs and the *Global strategy for women’s, children’s and adolescents’ health (2016–2030)*^6^ offer an opportunity for a more holistic and integrated approach to implementing and evaluating progress across sectors.

### Limitations

As part of the Success Factors studies, the countries in this analysis were first selected based on their accelerated progress towards the MDGs between 1990 and 2012. If the country selection had been later, for example after the final MDG progress reports in 2015, a different set of countries might have been identified. Nevertheless, these countries had made significantly better progress than other comparable countries at a specific point in time^4^^,^^5^ and this analysis highlights how they achieved this progress. The data synthesis was constrained by the data used for the dialogues. For example, several countries preferred to use national data over international data in the dialogues as it was perceived as more relevant and reliable for country-specific policies and programmes. This limited the ability to compare quantitative data across countries. The dialogues attempted to ensure that the inputs met basic criteria for plausibility. However, limited data in some areas sometimes made it difficult to meet all the plausibility criteria. Given the difficulties in quantifying the strength of individual policy and programme inputs and their relative contributions to improved health outcomes, we did not attempt to weight the identified strategies across countries. Thus, the impact of particular policies and programmes – the extent to which they were directly associated with observed health outcomes – was subject to interpretation, although we verified these where possible through data triangulation and consensus among stakeholders. The development of robust dialogue processes, including preparation, evidence review and explicit plausibility criteria, as described elsewhere,^7^ can help address some of these limitations. In all countries there is a need for better local data on country policy and programme inputs across sectors, and for better evaluation of the association of the inputs with overall health and sustainable development.

## Conclusion

Reducing maternal and child mortality in the 10 fast-track countries can be linked to consistent and coordinated policy and programme inputs across sectors. Approaches used by successful countries have relevance to other countries looking to scale-up or accelerate progress and can inform countries’ progress towards the SDGs.
